# Contrasting clinical evidence for market authorisation of cardio-vascular devices in Europe and the USA: a systematic analysis of 10 devices based on Austrian pre-reimbursement assessments

**DOI:** 10.1186/1471-2261-14-154

**Published:** 2014-11-04

**Authors:** Claudia Wild, Judit Erdös, Ingrid Zechmeister

**Affiliations:** Ludwig Boltzmann Institute for Health Technology Assessment (LBI-HTA), Garnisongasse 7/20, 1090 Vienna, Austria

**Keywords:** Approval, Market authorisation, Medical devices, Evidence based medicine/EbM, Health Technology Assessment/HTA, Cardio-vascular disease, Surgery, Safety

## Abstract

**Background:**

European medical device regulation is under scrutiny and will be re-regulated with stricter rules concerning requirements for clinical evidence for high-risk medical devices. It is the aim of this study to analyse the differences between Europe and USA in dealing with risks and benefits of new cardio-vascular devices.

**Methods:**

Since no information is available on clinical data used by the Notified Body for CE-marking, data from Austrian pre-reimbursement assessments close to European market approval were used as proxy and compared with clinical data available at time of market approval by FDA in the USA.

**Results:**

10 cardio-vascular interventions with 27 newly CE approved medical devices were analysed. The time lag between market authorisation in Europe and in the USA is 3 to 7 years. Only 7 CE-marked devices also hold a FDA market approval, 7 further devices are in FDA approved ongoing efficacy trials. For 4 of the CE-marked devices the FDA market application or the approval-trial was either suspended due to efficacy or safety concerns or the approval was denied. Evidence available at time of CE-marking are most often case-series or small feasibility RCTs, while large RCTs and only in rare cases prospective cohort studies are the basis of FDA approvals. Additionally, the FDA often requires post-approval studies for high-risk devices.

**Conclusions:**

Market authorisation based on mature clinical data deriving from larger RCTs and longer follow-ups do not only change the perspective on the risk-benefit ratio, but also secures real patient benefit and safety and assures payers of investing only in truly innovative devices.

## Background

Because of several market withdrawals due to unsafe or ineffective devices, European medical device regulation is under scrutiny and will be re-regulated with stricter rules concerning requirements for clinical evidence for class III (active) and class IIb (inactive) implantable medical devices [1]. The criticism from health care providers [2, 3] as well as from pre-coverage health technology assessors (HTA) and payers [4–6], but also from patient groups [5] is nurtured by the fact that unsafe devices reach the European markets with pre-mature clinical data. Re-regulation details are still under debate: It has been suggested to reduce the number of European market authorisation agencies (“Notified Bodies/NB”) from the current 75 to a few certified ones to approve highly specialised devices or to even go for complete centralisation, as is the case with drugs. Another suggestion is to transparently document the approval process, the evidence requirements and the provided clinical data [7].

It is well known that new medical devices, including all high-risk cardio-vascular devices, receive the European market approval (CE/ Conformité Européenne mark) several years prior to USA market authorisation [2]. Due to the lack of transparency *where* the devices are approved and *what* the decentralised NBs receive as application information, the actual clinical evidence can only be speculated via clinical studies published after the CE mark was issued.

Still, because of pressure from physician groups wishing to offer patients early access to innovative medicine and from manufacturers wishing early market expansion, applications for coverage of those newly CE marked devices are submitted to reimbursement institutions only several months later [8]. In a recent analysis of seven devices from all medical disciplines, it could be shown that Austria is among the first countries where applications for uptake and inclusion into the benefit catalogue are put forward [8]. Since 2008, the Ludwig Boltzmann Institute for HTA (LBI-HTA) has been commissioned by the Austrian Ministry of Health (MoH) to assess new hospital interventions, whereby new high-risk cardio-vascular implantable devices have accounted for one-third of all primary assessments.

The aim of this study is to analyse what evidence was available for cardio-vascular devices at the time of CE marking using the evidence presented in the Austrian assessments as a proxy, and to compare this data with the data available at the time of FDA (Food and Drug Administration) approval. Thereby, we intend to challenge the argument that earlier provision of new devices is always of benefit to the patient.

## Methods

Since no information is available on clinical data used by the NB for CE marking and because the Austrian pre-reimbursement assessments are rather close to CE marking, we assumed that the clinical evidence – provided by the manufacturer – used for CE mark could be considered a subset of the one used for the assessments produced for deciding on including the interventions in the hospital benefit catalogue. For this study we assumed the evidence is the same. Out of all pre-reimbursement assessments we selected those that dealt with cardio-vascular interventions. Since the intention of the analysis was to concentrate on the level of evidence of *new* medical devices, we excluded those assessments that dealt with a) interventions where no new (defined by us as max. 3–5 years prior to assessment) CE marked products were available at the time of assessment, b) expansion of established indications (new interventions) with already existing products, and c) cerebro-vascular interventions.

From the included assessments (published between 2006 and 2014) we extracted information on a) intervention, b) devices, c) indication and d) best available evidence in terms of number and types of studies and number of study participants. Furthermore, we searched for publicly available online information on CE marking (spring 2014) and, in cases of missing information, contacted the manufacturers directly, rather than consulting the NANDO (New Approach Notified and Designated Organisations) Database.

In the USA, new high-risk devices typically undergo a premarket approval (PMA) process based on an efficacy trial [9]. Devices can receive an IDE status (investigational device exemption) that allows the device to be used in clinical approval trials [10]. Moreover, by defining so-called HDE conditions (humanitarian device exemption), it is intended that patients with a disease manifested in fewer than 4,000 individuals benefit from a device [11]. To obtain information on the time of FDA approval and on available evidence at the time of approval, we searched for PMA, IDE, and HDE documents related to the selected interventions and devices (June to July 2014). Again, data on clinical evidence for PMA decisions and trial information were extracted.

Next, we assigned the levels of clinical evidence according to the Oxford Centre for Evidence-based Medicine hierarchy: 1: SR (systematic review) or MA (meta-analysis) based on several high quality RCTs (randomized clinical trials); 2: at least 1 RCT of high quality; 3: CTs (controlled trials) without randomisation; 4: prospective case–control and cohort studies; 5: case reports and retrospective case series [12].

Finally, we compared the market authorisation status and the level of evidence available around the time of market authorisation between Europe and the USA. Moreover, we analysed in more detail the differences between Europe and the USA in dealing with clinical evidence and with risk-benefit evaluations.

## Results

Between 2008 and 2014, 15 (out of 48) pre-reimbursement assessments dealt with cardio-vascular interventions. From those, assessments on 10 cardio-vascular interventions were finally included into the analysis according to the pre-defined criteria (Table 1).

**Table 1:** **Cardio-vascular interventions and medical devices, years of approval in Europe and in the USA, year of reimbursement-application in Austria (2008–2014)**

Intervention	Product/manufacturer	Indication	Year of CE mark by NB/notified body	Year of reimbursement application	Year of FDA approval	Time between EU and USA approval
1. Percutaneous pulmonary valve implantation for right ventricular outflow tract dysfunction in patients with congenital heart defects	Melody™/Medtronic	Patients born with a dysfunctional conduit of the right ventricular outflow tract (RVOT)	2006	2008	2010	4 years
					Jan: HDE Melody™	
2. Percutaneous aortic valve replacement/TAVI	Edwards SAPIEN XT™/Edwards Lifesciences	Inoperable or high-risk patients with severe aortic valve stenosis	2010	2008	2014	4 years
			2011	Updates: 2009, 2010, 2011	Jan: PMA	3 years
					CoreValve^®^	
	CoreValve^®^/Medtronic				June: PMA	
					Edwards SAPIEN XT™	
3. Cardiac contractility modulation (CCM) for heart failure	Optimizer™ III/Impulse Dynamics	Patients with symptomatic heart failure, NYHA Stadium ≥ II and normal QRC complex in ECG	2007	2008	No PMA	-
				Updates: 2009, 2010		
4. Percutaneous transluminal angioplasty (PTA) of *periphery* arteries with drug-eluting balloon (DEB)	In.Pact™ Amphirion + Admiral/Medtronic	Patients with peripheral artery diseases, including extracranial carotid and vertebral artery disease, upper extremity artery disease, mesenteric artery disease, renal artery disease as well as lower extremity artery disease	In.Pact™: 2009	2013	2014	5 years
			Lutonix DCB™:2011		July: Lutonix DCB™	
	Lutonix DCB™/Bard-Lutonix					
			Cotavance™: 2011			
	Cotavance™/Bayer Schering					
			Advance PTX^®^: 2011 LEGflow™: 2011/2012			
	Advance 18 PTX^®^/Cook Medical					
	LEGflow™/Cardionovum					
	ELUTAX SV™/Aachen Resonance		Elutax SV™: 2013			
			Freeway™: year not disclosed			
	Freeway™/Eurocor					
5. Percutaneous transluminal *coronary* angioplasty (PTCA) with drug-eluting balloon (DEB)	Dior^®^ PCB/Eurocor SeQuent^®^ Please DCB/Braun Melsungen AG	Patients with coronary artery diseases with in-stent-restenosis (ISR), ostium stenosis, stenosis of small coronary vessel disease (SVD) and de-novo lesion of coronary vessels	Dior^®^ PCB: 2007	2009	No PMA	-
			SeQuent^®^ Please DCB: 2009	Update: 2013		
			In.Pact™ Admiral: 2009			
	In.Pact™ Admiral/Medtronic					
	Cotavance™/Bayer Schering		Cotavance™: 2011			
6. Percutaneous repair of mitral regurgitation with the MitraClip	MitraClip^®^/Abbott	Patients with moderately severe or severe mitral regurgitation (grade 3+ or 4+); both operable and inoperable patients	2008	2010	2013	5 years
				Update: 2012	March: PMA	
					MitraClip^®^/Abbott	
7. Renal Denervation	Symplicity™ RDN/Medtronic	Patients with therapy resistant hypertonia after unsuccessful treatment (no blood pressure decrease) with at least 3 antihypertensive medicaments	2008	2011	No PMA	-
				Update: 2012		
8. Endovascular repair of aortic aneurysms	Zenith^®^ Fenestrated AAA Endovascular Graft/Cook Medical	Patients with abdominal aortic aneurysm and/or iliacal aneurysm having morphology suitable for endovascular repair	Zenith^®^ Fenestrated AAA Endovascular Graft: 2005	2013	2012	7 years
					Zenith^®^ Fenestrated AAA Endovascular Graft	
	Ventana™ Fenestrated System/Endologix		Ventana™ Fenestrated System: 2013			
9. Percutaneous transluminal angioplasty (PTA) with drug-eluting stents in peripheral arterial disease, upper limb and thorax	*Femoropopliteal*: CYPHER^®^ Select/Cordis	Patients with symptomatic peripheral artery disease (PAD) on arteria femoralis superficialis/SFA, below the knee/BTK, visceral arteria, iliacalarteria, upper extremities	CYPHER^®^ Select: 2005 Zilver^®^ PTX^®^: 2009	2014	2012	3 years
					Zilver^®^ PTX^®^	- - -
	Zilver^®^ PTX^®^/Cook Medical					
			Innova™: 2012			
	Innova™/Boston Scientific					
			S.M.A.R.T.^®^: 2013 XIENCE Prime BTK: 2011			
	S.M.A.R.T.^®^/Cordis					
	*Infrapopliteal*:		Yukon^®^: 2011			
			PROMUS Element™ Plus			
	XIENCE^®^ Prime BTK/Abbott					
			DES BTK: 2012			
	Yukon^®^/Translumina					
	PROMUS Element™Plus					
	DES BTK/Boston Scientific					
10. Percutaneous left atrial appendage closure for the prevention of thromboembolic events in patients with atrial fibrillation	Watchman^®^ LAA Closure Technology/Atritech-Boston Scientific	Patients with atrial fibrillation (AF)/flutter/cardiac arrhythmia/abnormal heart rhythm to prevent thromboembolic events such as ischaemic stroke	Watchman^®^ LAA: 2005, 2012 (extended use)	2011	No PMA	- - -
				Update: 2014		
	AMPLATZER™ Cardiac Plug/St. Jude Medical		AMPLATZER^TM^ Cardiac Plug: 2008			
			WaveCrest™: 2013			
	Coherex WaveCrest™ LAA Occlusion System/Coherex Medical					

### Market authorisation granted

For the 10 cardio-vascular interventions analysed, 27 newly approved CE marked medical devices were available (see details in Table 2). Of those, only 6 devices also hold a PMA status, 1 holds the HDE status, while 7 medical devices are under IDE. For 4 of the devices, the application in the USA was either suspended due to efficacy or safety concerns before/during/after the IDE trials (Cotovance™, Ventana™, Symplicity™), or market authorisation was denied (Watchman^®^) due to safety concerns. 12 CE marked cardio-vascular devices are neither PMA-approved, nor hold an IDE status (yet), meaning that they are produced solely for the European market or the IDE will be applied for at a later stage. For 1 device (CYPHER^®^ Select), marketing was discontinued in Europe.

**Table 2:** **Evidence available at time of pre-reimbursement assessment in Austria and at time of FDA approval, levels of evidence**

Intervention	Product	Highest level of evidence at time of pre-reimbursement assessment (Austria)	LoE	Highest level of evidence: FDA application/approval (USA)	LoE
1. Percutaneous pulmonary valve implantation for right ventricular outflow tract dysfunction in patients with congenital heart defects	Melody™	NB 2006		FDA 2010	4
		2008 [14]: 4 retrospective + prospective case series, 8–68 pts	5	HDE approval based on 1 prospective case series 99 pts, requirement of 2 post-approval studies with 5y FU [13]	
2. Percutaneous aortic valve replacement/ TAVI	CoreValve^®^	2007		FDA 2014	2
	SAPIEN XT™	2008 [16]: retrospective + prospective 10 case series, 8–86 pts	5	CoreValve^®^ PMA approval based on 1 RCT, 656 pts [15] (CoreValve U.S. Pivotal Trial), requirement for post-approval study on extreme risk patients.	
		2009 [18]: see 2008 + 4 case series, 12–646 pts 2010 [19]: see 2008 + 2009 + 6 cohort studies (registries), 4 HTAs	4		
			4	SAPIEN XT™ PMA approval based on 1 RCT, 560 pts [17] (PARTNER II), requirement for post-approval study on inoperable patients cohort	2
			2		
		2011 [20]: see 2008 + 2009 + 2010 + 1 RCT, 358 pts (PARTNER I)			
3. Cardiac contractility modulation (CCM) for heart failure	Optimizer™ III/IV	NB 2007		-	-
		2008 [21]: 2 RCTs, 49/164 pts (FIX-HF-4/ OPTIMIZER) +2 case series 13/25 pts)	2	*IDE Status:* Optimizer™ III Optimizer™ III/IV Trial FIX-HF-5 (Evaluate Safety and Efficacy of the OPTIMIZER^®^) ongoing	
		2009 [22]: see 2008 + 1 RCT-Protokoll, 428 pts (FIX-HF-5/ OPTIMIZER)	2		
		2010 [23]: see 2009	2		
4. Percutaneous transluminal angioplasty (PTA) of *periphery* arteries with drug-eluting balloon (DEB)	In.Pact™ Amphirion + Admiral	NB 2009/11/12/13		FDA 2014	1 + 3
		2013 [25]: 4 RCTs, 50–102 pts(2 × In.Pact™ DEBELLUM, PACIFIER, 2 × Cotovance™ THUNDER, FemPac) +2 cohort studies (registries: In.Pact™ Admiral, In.Pact™ Amphirion)	1-2	Lutonix DCB^®^ PMA based on LEVANT 2 RCT, 476 pts + LEVANT 2 registry, 657 pts. [24]	
	Lutonix DCB^®^				
	Cotavance™				
	Advance 18 PTX^®^			*IDE Status*: In.Pact™ Admiral based on SFA I and SFA II RCT, 331 pts	
	LEGflow™			*No IDE Status*: Cotavance™ (IDE suspended), Advance 18 PTX (only EU), LEGflow™ (RAPID trial EU),Freeway™ (only EU)	
	ELUTAX SV™				
	Freeway™				
5. Percutaneous transluminal *coronary* angioplasty (PTCA) with drug-eluting balloon (DEB)	Dior^®^ PCBr	NB 2007/09/11		-	-
	SeQuent^®^ DCB In.Pact™ Admiral	2009 [26]: 2 RCTs, 52/108 pts (2x Cotavance™ PACCOCATH I, II)	1-2	*IDE Status*: In.Pact™ Admiral/ Medtronic based on SFA I and SFA II RCT, 331 pts	
	Cotavance™	2013 [27]: 5 RCTs for ISR, 50–271 pts (4 × SeQuent^®^ PEPCAD II-IV + 1 × Cotavance™ PACCOCATH II FU) +1 RCT for SVD, 60 pts (Dior^®^ PICCOLETO) +1 RCT for de-novo lesions, 84 pts (SeQuent^®^)	1-2	*No IDE Status* :Dior^®^ PCB (only EU), SeQuent^®^ DCB: (only EU), Cotavance™ (IDE suspended)	
6. Percutaneous repair of mitral regurgitation with the MitraClip	MitraClip^®^/Abbott	NB 2008	4	FDA 2013	
		2010 [28]: prospective case series (EVEREST I/II), 107 pts 2012 [30]: 2010 + 10 case series +1 RCT, 279 pts (EVEREST II)	2	MitraClip^®^ PMA approval based on EVEREST II RCT, 279 pts., EVEREST II high-risk registry (EVEREST II HRR), 78 pts and EVEREST II Cont. access registry (REALISM HR), 853 pts [29], requirement of 2 post-approval studies (registries)	2 + 3
7. Renal denervation	Symplicity™ RDN/Medtronic	NB 2008		-	-
		2011 [31]: 1 prospective case series (SYMPLICITY HTN-1), 50 pts; 1 RCT, 106 pts. (SYMPLICITY HTN-2)	4, 2	*IDE Status*: IDE approval study SYMPLICITY HTN-3, 535 pts. failed; 2014 approval process suspended [32], IDE approval study SYMPLICITY HTN-4 ongoing	
			4, 2		
		2012 [33]: 2011+ 1 case series, 153 pts. (FU SYMPLICITY HTN-1)			
8. Endovascular repair of aortic aneurysms	Zenith^®^ Fenestrated AAA Endovascular Graft	NB 2005/13		FDA 2012	4
		2013 [34]: 2 HTAs +4 SR based on 1 (non-randomised) controlled study, 187 pts +7-20 case series, 196–368 pts	3	Zenith^®^ Fenestrated AAA Endovascular Graft PMA approval based on 1 (historic case-) controlled study, 42 pts [35], requirement of a long-term follow-up study.	
	Ventana™ Fenestrated System				
				*IDE Status*: Ventana™ Fenestrated System	
9. Percutaneous transluminal angioplasty (PTA) with drug-eluting stents in peripheral arterial disease, upper limb and thorax	*Femoropopliteal*:	NB 2009/ 12/13	1 + 2 1 + 2	FDA 2012 Zilver^®^ PTX^®^ PMA approval based on Zilver PTX randomised study, 479 pts. [37]	2
	Zilver^®^ PTX^®^ Innova™	2014 [36]: *Femoropopliteal*			
	S.M.A.R.T.^®^	3 RCTs, 36–479 pts (1 × Zilver^®^ PTX study, 2 × S.M.A.R.T.^®^ SCIROCCO I + II) +1 non-randomised CTs, 93 pts (Zilver^®^) +1 case series, 787 pts.		IDE Status: S.M.A.R.T.^®^ Vascular Stent System No IDE Status: Innova™ Peripheral Vascular DES System (MAJESTIC in Australia, New Zealand, EU); XIENCE^®^ Prime BTK;	
	*Infrapopliteal*:				
	XIENCE^®^ Prime BTK	*Infrapopliteal*		Yukon^®^; PROMUS Element™PlusDES BTK; CYPHER^®^ Select (discontinued to be marketed)	
	Yukon^®^	4 RCTs, 50–200 pts (1 × XIENCE^®^ DESTINY, 1 × Yukon^®^ YUKON-BTK, 2× CYPHER^®^ ACHILLES) +3 non-randomised CTs, 58–103 pts +3 case series, 114–146 pts (all CYPHER^®^ Select)			
	PROMUS Element™PlusDES BTK				
	CYPHER^®^ Select				
10. Percutaneous left atrial appendage closure for the prevention of thromboembolic events in patients with atrial fibrillation	Watchman^®^ LAA Closure Technology	NB 2005/08/12/13	4 + 5	-	-
		2011 [38]: 4 case series, 64–180 pts; 1 cohort study/ registry, 73 pts; 1 RCT, 542 pts (Watchman^®^ PROTECT AF)		2009: Watchman^®^ LAA Closure Technology PMA approval based on RCT, 707 pts (PROTECT AF) *declined* [39]	
	AMPLATZER™ Cardiac Plug				
			3 + 2		
	Coherex WaveCrest™ LAA Occlusion System	2014 [40]: 2011 + 3 FU studies of PROTECT AF +		*IDE Status*: Watchman^®^ LAA Closure, re-application with RCT PREVAIL, 407 pts; AMPLATZER™ Cardiac Plug (RESPECT), 980 pts	
		3 case series, 52–86 pts +1 cohort study/ registry, n/a; 2 prospective CTs, 80/150 pts			

Legend: pts.: patients; NB: notified bodies; RCT: randomised controlled trial; FDA: Food and Drug Administration; IDE: investigational device exemption; PMA: premarket approval; HDE: humanitarian device exemption.

### Time lag in market authorisation

For those 7 (6: PMA; 1: HDE) cardio-devices holding European and US licences, the time lag between market authorisation in Europe and in the USA amounted to 3 to 7 years.

### Levels of evidence

The applications for coverage of new interventions and devices in cardio-vascular pathologies were submitted between 1 to 3 years after CE marking. In most of the 10 analysed interventions, the evidence available at the time of pre-reimbursement assessment was either level 4–5 (retrospective, sometimes prospective case series without control groups) or 2 (small RCTs/feasibility studies for individual devices/1^st^ generation). Non-randomised controlled studies (level 3) or prospectively planned registries (level 4) were seldom available. On the contrary, IDE-accepted RCTs (level 2) or – in some rare medical conditions – prospective case series or cohort studies (level 3 and 4) were the basis for FDA approval decisions in most cases (Table 2).

A detailed analysis of the efficacy and safety assessments of individual devices seems to be more informative than the levels of evidence per se. Several differences in dealing with the evidence on benefit-risk ratios could be identified and the following patterns have appeared:

1. *Requirement of extensive follow-up for high-risk devices*: Melody™ was CE marked in 2006 and has held an HDE approval since 2010. An HDE application is *not* required to contain the final results of scientifically valid clinical investigations, but must contain sufficient information to determine that the device does not pose an unreasonable or significant risk and that the probable benefit to health outweighs the risks [13]. Therefore, two additional post-approval studies (150 patients, follow-up of 5 years and 100 new patients with primary analysis performed at 6 months) are required by the FDA for Melody™ [41]; these studies are ongoing. No such requirements exist in Europe, since registries are voluntary. Edwards SAPIEN XT™ and CoreValve^®^ received a CE mark in 2010 and 2011, and a PMA in 2014. The latter is based on RCTs with 560 (SAPIEN XT™) and 656 patients (CoreValve^®^) respectively. This type of clinical data was not available at the time of European market authorisation. Follow-up data from post-approval studies on inoperable and extreme risk patients is required by the FDA for both devices. No such requirements were defined at the time of EU market authorisation.
2. *Early approval in Europe and later demonstration of inefficacy in RCT*: Symplicity™ received CE marking in 2008 based on a safety study (SYMPLICITY HTN-1). In 2014, the FDA approval study (SYMPLICITY HTN-3) failed to meet its primary efficacy endpoint [42]. As a consequence, the manufacturer is considering a suspension of enrolment in the already ongoing SYMPLICITY HTN-4 (IDE) trial.
3. *Early approvals in Europe and safety concerns in the USA:* Three different devices were approved for percutaneous left atrial appendage closure for the prevention of thromboembolic events in patients with atrial fibrillation in Europe between 2005 and 2013; none of them holds a PMA status. WATCHMAN^®^ LAA Closure Technology was denied PMA in 2009 due to safety concerns. The FDA Circulatory System Devices Panel concluded (7 in favour, 5 opposed) that although short-term efficacy had been demonstrated by the data available from the PROTECT AF trial, longer term efficacy had not been adequately demonstrated due to the lack of available long-term data [39]. In late 2013, the PREVAIL data was presented; a PMA decision is pending [43].
4. *Devices of unknown value*: Optimizer*™* received the CE mark in 2007 based on a feasibility trial (showing no improvement in primary endpoints [21–23] and proceeded on to the IDE-approved FIX-HF-5 trial that has been running since 2011. Results are to be expected in 2015.
  For PTA (percutaneous transluminal angioplasty) of peripheral arteries with drug- eluting balloon (DEB), 6 devices received the CE mark between 2009 and 2013. Only one of these (Lutonix DCB™) also received PMA in 2014; another (In.Pact™ Admiral IDE) is expected to be approved by the FDA in 2015 or later. One of the DEBs, *Cotavance™,* is CE marked for the treatment of peripheral arterial disease (PAD), as well as stenotic lesions in the iliac and infrainguinal arteries, but the IDE application was suspended in 2012 due to problems concerning drug adhesion to the balloon and the according safety concerns [44].
5. *Devices of critical risk-benefit-ratio:* MitraClip^®^ received CE marking in 2008 on the basis of case series (EVEREST I); PMA followed in 2013. Although an RCT (EVEREST II), a prospectively planned registry (EVEREST II HRR) and a “Real World Expanded Multi-center Study” (REALISM HR) were submitted, FDA approval was cautious, with 5 votes to 3 on whether the benefits outweigh the risks and 4 votes to 5 on whether there is a reasonable assurance of efficacy.

Two devices for the endovascular repair of aortic aneurysms received CE marking in 2005 and 2013. Only one is also approved in the USA (2012); the other (Ventana™) holds an IDE, but the trial was suspended and enrolment was stopped because of a higher than expected number of re-interventions [45].

## Discussion

Recent publications showed that cardiovascular devices receiving PMA (between 2000 and 2007) are often (63%) based on non-randomised studies that lack adequate strength and may be prone to bias [46], that effectiveness endpoints are more often reported than safety endpoints, and that patient comorbidities are only incompletely reported [47]. Recalls are not uncommon, especially for those devices that have been cleared via the “substantial equivalence” process [48]. The US watchdog institution Public Citizen called medical devices in the USA “substantially unsafe” [49]. In contrast, the European debate is being led – with the exemption of the detailed analysis in [2] – on a much more general level. There is only a general demand for stricter regulation, since no data are available for analysis and there is a lack of transparency concerning *which* NB gave market authorisation on the basis of *what* type of clinical evidence.

Because of the earlier market authorisation and the lack of requirements other than the performance evaluation of medical devices in Europe (the lack of a definition of “performance” is resulting in totally different interpretations), the clinical evidence available at the time of pre-reimbursement assessments is naturally lower than some years later for market authorisation in the USA [5, 50, 51]. Several unsafe and ineffective devices are approved in the EU, but not in the USA [52]. The perspective of manufacturers (and of some clinical experts) that early market access provides highly innovative medicine to suffering patients [53] is held against the perspective that little is known on the effectiveness and on the risk-benefit ratio at the time of European market authorisation. Patients are put at risk and health care systems are put under pressure to invest in interventions of unknown value [6]. Since some cardio-vascular devices are seemingly intended for the non-US market only, the uncertainty on their benefits and potential harms may never be resolved.

Based on our experiences from 7 years of pre-reimbursement assessments [54], our study contributes to the existing knowledge that not only earlier approvals are based on limited data, but that more mature data deriving from larger randomised trials and longer follow-ups might also change the perspective on the risk-benefit ratio entirely. The two most obvious examples of devices that have been considered for market approval on both continents and where different conclusions were drawn are *Symplicity™* and *Watchman*^*®*^*.* Others are less visible, but still there: For *Optimizer™* (CE marked in 2007), no convincing evidence on efficacy has been demonstrated so far. In the case of *Cotavance™* (CE marked in 2011), steps towards FDA approval were suspended due to problems concerning drug adhesion to the balloon. For *Ventana™* (CE marked 2013), enrolment into a trial was stopped because of a higher than expected number of re-interventions. There may even be more examples we are not aware of. Further general deficiencies are well described by Fraser et al. [2].

This study has shortcomings: The biggest limitation is the fact that no data on the clinical evidence for the CE marking are available; we therefore had to rely on the information on available clinical studies derived from the Austrian pre-reimbursement assessments. Nevertheless, we think it is plausible that less rather than more clinical data were available at an earlier stage, though probably not all published. Additionally, only the devices for specific indications mentioned in the pre-reimbursement applications were assessed, meaning that other cardio-devices posing problems after CE marking such as ProRhytm^®^ and HD Mesh Ablator^®^ for the treatment of Atrial Fibrillation (AF) were not included in this analysis.

## Conclusions

Our conclusions are that good clinical evidence at the time of market authorisation not only secures real patient benefit and safety, but also assures payers of investing only in truly innovative devices. In addition, good clinical evidence might ease market access for manufacturers and make coverage in (hospital) benefit catalogues more predictable. There is a strong need for stricter device regulation in Europe and compulsory, long-term follow-up in order not to expose European patients to (often) premature experimental devices.

## Authors’ information

CW/Claudia Wild is director of the LBI-HTA and trained in communications and social medicine; IZ/Ingrid Zechmeister is deputy director of the LBI-HTA and trained in health economics; JE/Judit Erdos is research assistant at LBI-HTA and trained in International Health Care Management.

## Abbreviations

AF: Atrial fibrillation
CE: Conformité Européenne
CT: Controlled trials
FDA: Food and Drug Administration
HDE: Humanitarian device exemption
IDE: Investigational device exemption
LBI-HTA: Ludwig Boltzmann Institute for HTA
MA: Meta-analysis
MoH: Ministry of health
NANDO: New approach notified and designated organisations
PAD: Peripheral arterial disease
PMA: Premarket approval
PTA: Percutaneous transluminal angioplasty
RCT: Randomized clinical trial
SR: Systematic review
USA: United States of America.
